# Clinical significance of the Naples prognostic score in predicting short‐ and long‐term postoperative outcomes of patients with hepatocellular carcinoma

**DOI:** 10.1002/wjs.12448

**Published:** 2024-12-04

**Authors:** Kiyotaka Hosoda, Akira Shimizu, Koji Kubota, Tsuyoshi Notake, Noriyuki Kitagawa, Takahiro Yoshizawa, Hiroki Sakai, Hikaru Hayashi, Koya Yasukawa, Yuji Soejima

**Affiliations:** ^1^ Division of Gastroenterological, Hepato‐Biliary‐Pancreatic, Transplantation, and Pediatric Surgery Department of Surgery Shinshu University School of Medicine Matsumoto Japan

**Keywords:** hepatocellular carcinoma, outcome, postoperative complication, prognosis, risk factor

## Abstract

**Background:**

The Naples prognostic score (NPS) is a remarkable marker of short‐ and long‐term outcomes in various types of cancer. However, its impact on the postoperative outcomes of hepatocellular carcinoma remains controversial. This study aimed to clarify the impact of the NPS on the prognosis and incidence of postoperative complications in hepatocellular carcinoma.

**Methods:**

Patients with hepatocellular carcinoma (*n* = 374) were categorized into high‐ and low‐Naples prognostic score groups; their postoperative outcomes were compared. Prognostic and risk factors for severe postoperative complications were identified using multivariate analyses.

**Results:**

The low‐Naples prognostic score group had significantly longer overall and recurrence‐free survivals than the high‐Naples prognostic score group (*p* = 0.03 and 0.04, respectively). Subgroup analysis revealed a superior predictive value of the NPS in the group with a single tumor (*p* = 0.03), tumor diameter ≤5 cm (*p* = 0.04), and tumor stage I or II (*p* = 0.04). A high NPS was an independent prognostic factor for overall survival (hazard ratio, 1.45; 95% confidence interval (CI), 1.01–2.05; and *p* = 0.04). The NPS 2–4 group had a higher incidence of the Clavien–Dindo grade ≥ IIIa postoperative complications than the 0–1 group (*p* = 0.03) and a score of 2–4 was identified as an independent risk factor for the Clavien–Dindo grade ≥ IIIa postoperative complications (odds ratio, 2.06; 95% CI, 1.01–4.20; and *p* = 0.05).

**Conclusions:**

The NPS effectively predicts postoperative outcomes in patients with hepatocellular carcinoma.

## 1 INTRODUCTION

Liver cancer is the sixth most common cancer and the second leading cause of cancer mortality worldwide according to the GLOBOCAN 2020.^1^ Hepatocellular carcinoma (HCC) is the most common liver cancer that develops in hepatocytes owing to chronic inflammatory liver diseases.^2^ Although various treatment options exist for HCC, including surgery and chemotherapy, the recurrence rate is high leading to a dismal prognosis.^3^ Hepatectomy is the first‐choice therapy for HCC; however, it can lead to posthepatectomy liver failure (PHLF) and other severe complications that are not always predictable preoperatively.

Previous studies have revealed that inflammation‐based prognostic scores (IBPSs), such as neutrophil‐to‐lymphocyte ratio (NLR), platelet‐to‐lymphocyte ratio, and lymphocyte‐to‐monocyte ratio (LMR), could be markers of outcomes of HCC surgeries.^4^, ^5^, ^6^ Recently, new markers based on the IBPSs or nutritional indices have been established, among which the Naples prognostic score (NPS) has been garnering significant attention. The NPS is evaluated based on serum albumin, total cholesterol (TC) levels, NLR, and LMR and reflects inflammation, malnutrition, and immunosuppression.^7^, ^8^, ^9^ Moreover, the NPS has been reported to be a remarkable marker of short‐ and long‐term outcomes in various types of cancers.^7^, ^8^, ^9^, ^10^, ^11^, ^12^, ^13^, ^14^, ^15^ However, studies focusing on the relationship between postoperative outcomes or prognosis after surgery for HCC and NPS are rare.^9^

This study aimed to clarify the impact of NPS on the prognosis and incidence of postoperative complications in HCC.

## 2 METHODS

### 2.1 Patients

**FIGURE 1:**
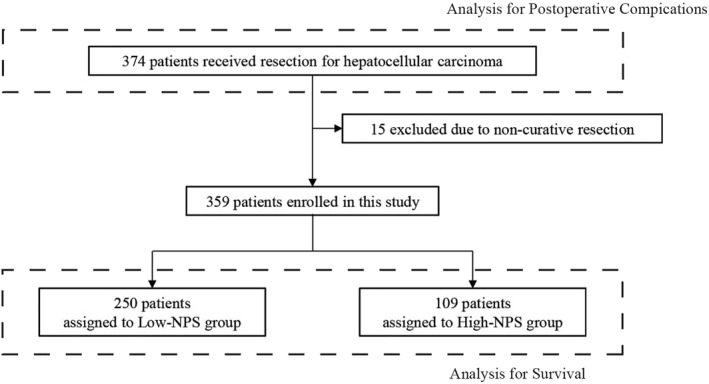
Patient selection criteria flowchart. NPS, Naples prognostic score.

This research was conducted at the Division of Gastroenterological, Hepato‐Biliary‐Pancreatic, Transplantat‐ion, and Pediatric Surgery, Department of Surgery, Shinshu University Hospital, Japan. We retrospectively analyzed 374 patients who underwent initial hepatectomy for HCC between January 2007 and December 2021. Fifteen patients who underwent noncurative resections were excluded from the survival analysis (Figure 1).

The protocol for this research project has been approved by a suitably constituted Ethics Committee of the institution and it conforms to the provisions of the Declaration of Helsinki (the Shinshu University Hospital Ethics Committee, Approval No. 5244). An opt‐out procedure was applied to the website to obtain informed consent and patients who refused to participate were excluded.

### 2.2 Data collection and endpoint assessment

Clinicopathological data were retrospectively extracted from medical records and analyzed. Preoperative laboratory data were obtained 1–3 days prior to surgery. The NPS was assessed using serum albumin, TC levels, NLR, and LMR as previously described by Galizia G et al^10^; a score of 0 or 1 was given with reference to the cutoff value (serum albumin ≥4.0 g/dl, TC ≥ 180 mg/dl, NLR <2.96, and LMR ≥4.44) for each item and the sum of the scores was calculated. The participants were divided into three groups; participants with scores of 0, 1–2, and 3–4 were assigned to groups 0, 1, and 2, respectively. Survival was compared between the low‐ (NPS group 0–1) and high‐ (NPS group 2) NPS groups in the entire cohort and subgroups, and prognostic factors were identified using multivariate analysis. The final pathological stage was identified using the eighth edition of the *TNM Classification of Malignant Tumors* by the Union for International Cancer Control (UICC). Postoperative complications were assessed using the Clavien–Dindo classification (CD).^16^ The incidence of complications was compared between the two groups classified by the NPS values and risk factors were identified.

### 2.3 Perioperative management and surgical procedure

The indications for hepatectomy were evaluated based on the Makuuchi criteria.^17^ The extent of hepatectomy was moderated in accordance with tumor localization and various scores indicating liver function or fibrosis (e.g., serum levels of type IV collagen 7s domain 18 and the remnant hepatocellular uptake index^19^).

Postoperative surveillance was performed as previously described.^20^

### 2.4 Statistical analysis

Comparative evaluations between the high‐ and low‐NPS groups were undertaken with the χ^2^ test or Fisher's exact test for categorical attributes and the Wilcoxon rank sum test for quantitative attributes. Categorical variables were summarized as number and relative proportions and continuous variables as median values and ranges. The Kaplan–Meier method was used to analyze overall survival (OS) and recurrence‐free survival (RFS), and the statistical significance of the survival curves was analyzed using the log‐rank test. Median survival time (MST) was defined as the median OS or RFS. Subgroup analysis of the OS was performed to explore the predictive value of the NPS for distinct populations. The results of the subgroup analyses are summarized using a forest plot. Univariate and multivariate analyses for prognostic and risk factors for postoperative complications were performed using the Cox proportional hazards and multiple logistic regression models, respectively. Hazard ratios (HRs) and odds ratios (ORs) with 95% confidence intervals (CIs) were also calculated and presented. *p*‐values <0.05 were considered statistically significant. All statistical analyses were performed using JMP version 13.2.1 (SAS Institute Inc., Cary, NC, USA).

## 3 RESULTS

### 3.1 Baseline demographic and clinical characteristics

The baseline demographic and clinicopathological features of the patients are summarized in Table 1. The NPS of the entire cohort was as follows: group 0, *n* = 40 (11.1%); group 1, *n* = 210 (58.5%); and group 2, *n* = 109 (30.4%). In the patients' baseline demographics, age (*p* < 0.01), rate of alcoholic liver disease (*p* < 0.01), and preoperative serum level of total bilirubin (*p* < 0.01) of the high‐NPS group were significantly higher than those of the low‐NPS group; operative findings were comparable between groups. The final pathology showed a significantly larger tumor diameter (*p* = 0.01) in the high‐NPS group, whereas the UICC TNM staging did not show a significant difference between the high‐ and low‐NPS groups.

**TABLE 1:** Patients' background, operative findings, and pathological findings.

	Low‐NPS		High‐NPS		
Variables	*n* = 250		*n* = 109		*p* Value
Age^a^, year	70	(16–85)	73	(44–89)	0.001^b^
Sex					0.472
Male	188	(75.2)	78	(71.6)	
Female	62	(24.8)	31	(28.4)	
Body mass index^a^, kg/m^2^	22.9	(13.4–45.2)	22.4	(16.2–34.7)	0.214
Underlying liver disease					
Hepatitis B virus	105	(42.0)	49	(45.0)	0.603
Hepatitis C virus	95	(38.0)	51	(46.8)	0.120
Alcohol	39	(15.6)	6	(5.5)	0.005^b^
NASH/NAFLD	19	(7.6)	6	(5.5)	0.464
Liver cirrhosis	37	(33.9)	77	(30.8)	0.557
Diabetes mellitus	88	(35.2)	34	(31.2)	0.459
Total bilirubin^a^, mg/dl	0.84	(0.28–2.81)	0.71	(0.15–2.08)	<0.001^b^
Indocyanine green retention rate at 15 min^a^, %	12.0	(2.7–46.4)	12.9	(2.6–89.0)	0.630
Alfa‐fetoprotein^a^, ng/mL	8.0	(0.7–186800)	9.3	(1.1–54059)	0.442
Operative procedure					0.245
Hemihepatectomy	22	(8.8)	17	(16.0)	
Sectionectomy	49	(19.6)	22	(20.2)	
Segmentectomy	84	(33.6)	30	(27.5)	
Partial	95	(38.0)	40	(36.7)	
Operation time^a^, min	344	(82–990)	353	(121–694)	0.157
Blood loss^a^, ml	300	(0–5100)	350	(0–6600)	0.085
Blood transfusion	30	(12.0)	21	(19.3)	0.076
Tumor diameter^a^, cm	2.9	(0.9–17.0)	3.5	(1.3–16.5)	0.008^b^
Multiple tumors	60	(24.0)	25	(22.9)	0.827
Vascular invasion					
Portal vein invasion	56	(22.4)	33	(30.6)	0.106
Hepatic vein invasion	26	(10.4)	19	(17.6)	0.066
UICC Staging, 8th edition					0.180
IA	55	(22.4)	13	(12.0)	
IB	87	(34.8)	38	(35.2)	
II	77	(30.8)	41	(38.0)	
IIIA	14	(5.6)	9	(8.3)	
IIIB	16	(6.4)	7	(6.5)	

*Note*: Figures in parentheses are percentage unless otherwise specified.

Abbreviations: NAFLD, nonalcoholic fatty liver disease; NASH, nonalcoholic steatohepatitis; NPS, Naples prognostic score; UICC, Union for International Cancer Control.

^a^ median (range).

^b^ *p* < 0.05.

### 3.2 Survival analysis

**FIGURE 2:**
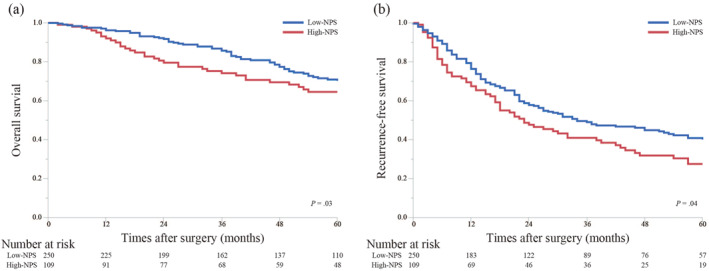
Kaplan–Meier curves for the overall survival (OS) and recurrence‐free survival (RFS) of the participants stratified by high‐ and low‐NPS groups. The OS (A) and RFS (B) of the high‐NPS group are significantly lower than that of the low‐NPS group (OS, *p* = 0.03 and RFS, *p* = 0.04). NPS, Naples prognostic score; OS, overall survival; RFS, recurrence‐free survival.

The OS and RFS of the low‐NPS group were significantly longer than those of the high‐NPS group (MST [OS], 108 vs. 92 months, *p* = 0.03; Figure 2B and MST [RFS], 34 vs. 23 months, *p* = 0.04; Figure 2B, respectively).

**FIGURE 3:**
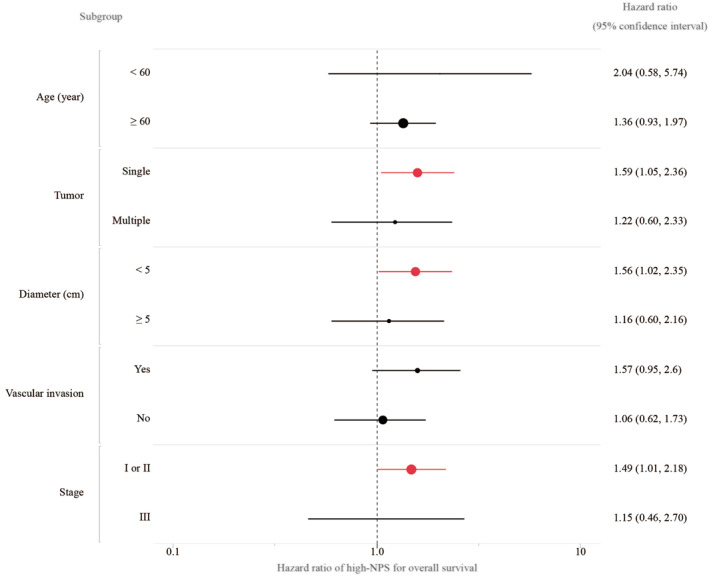
Subgroup analysis for the survival predictability of the Naples prognostic score (NPS). A high NPS is a significant prognostic factor for overall survival in patients with single tumor (*p* = 0.03), small tumor (diameter <5 cm) (*p* = 0.04), and tumor stage I or II (*p* = 0.04). NPS, Naples prognostic score.

Subgroup analysis revealed that a high NPS was significantly associated with poor OS in patients' subgroups of single tumor (HR, 1.59; 95% CI, 1.05–2.39; and *p* = 0.03), small tumor (diameter ≤5 cm) (HR, 1.55; 95% CI, 1.02–2.35; and *p* = 0.04), and UICC stage I or II (HR, 1.49; 95% CI, 1.01–2.18; and *p* = 0.04) (Figure 3). Moreover, OS and RFS were significantly longer than those of the high‐NPS group for patients with a single tumor smaller than 5 cm (MST [OS], 134 vs. 105 months, *p* = 0.01; Figure S1a and MST [RFS], 54 vs. 27 months, *p* = 0.02; Figure S1b, respectively).

### 3.3 Analysis of prognostic factors

Univariate and multivariate analyses of the OS were performed to explore the prognostic factors. In the univariate analysis, multiple tumors (*p* = 0.03), tumor diameter >5 cm (*p* < 0.01), portal vein invasion (*p* < 0.01), alpha‐fetoprotein level >10 ng/ml (*p* = 0.01), and high NPS (*p* = 0.04) were significantly associated with poor OS (Table 2). Multivariate analysis using the Cox proportional hazards model with variables with significant differences in the univariate analysis showed portal vein invasion (HR, 2.43; 95% CI, 1.68–3.48; and *p* < 0.01), multiple tumors (HR, 1.51; 95% CI, 1.04–2.18; and *p* = 0.03), and high NPS (HR, 1.45; 95% CI, 1.02–2.05; and *p* = 0.04) as the independent prognostic factors for the OS (Table 2). The multivariate analysis for RFS also revealed a high NPS as one of the independent prognostic factors (HR, 1.33; 95% CI, 1.00–2.52; *p* = 0.05; and Table S1).

**TABLE 2:** Univariate and multivariate analyses of prognostic factors for overall survival.

Variables	Univariate			Multivariate		
	Hazard ratio	95% CI	*p* Value	Hazard ratio	95% CI	*p* Value
Tumor diameter						
<5 cm	Ref			Ref		
≥5 cm	1.67	1.25–2.40	0.008^a^	1.28	0.87–1.86	0.206
Multiple tumor						
No	Ref			Ref		
Yes	1.52	1.04–2.18	0.030^a^	1.51	1.04–2.18	0.031^a^
Alpha‐fetoprotein						
<10 ng/mL	Ref			Ref		
≥10 ng/mL	1.53	1.10–2.16	0.013^a^	1.20	0.84–1.70	0.317
Portal vein invasion						
No	Ref			Ref		
Yes	2.70	1.91–3.80	<0.001^a^	2.43	1.68–3.48	<0.001^a^
NPS						
Low‐NPS	Ref			Ref		
High‐NPS	1.45	1.02–2.05	0.040^a^	1.45	1.02–2.05	0.041^a^

Abbreviations: CI, confidence interval; ICG, indocyanine green; NPS, Naples prognostic score.

^a^ *p* < 0.05.

### 3.4 Assessment of postoperative complications

The postoperative mortality rate was 0.3% (*n* = 1), and the morbidity rates of CD all grade and CD grade ≥ IIIa complications were 56.1% (*n* = 210) and 18.4% (*n* = 69), respectively.

**FIGURE 4:**
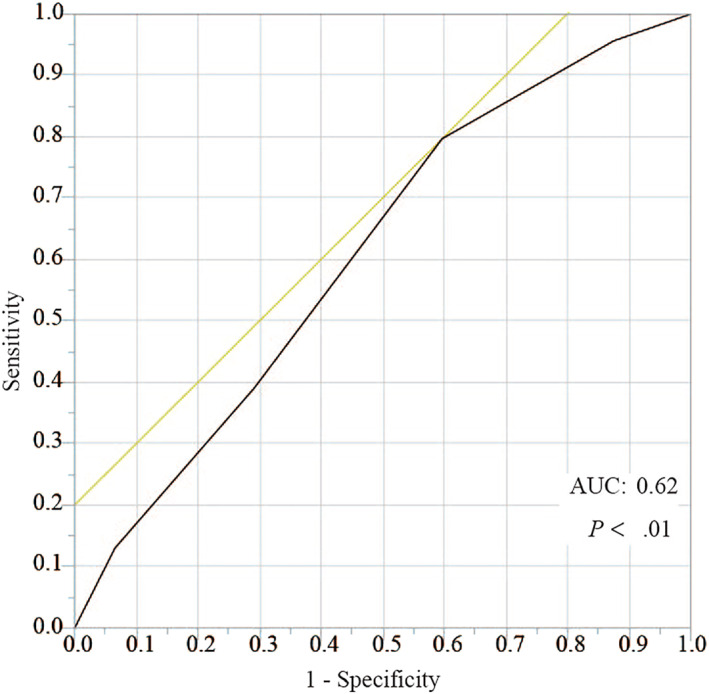
Receiver operating characteristic curve analysis of the Naples prognostic score (NPS) for postoperative complications with CD grade ≥ IIIa. The area under the curve value is 0.62 and the optimal cutoff value is NPS score 2. CD, Clavien–Dindo classification; NPS, Naples prognostic score.

According to the cutoff value of the NPS, determined using the receiver operating characteristic (ROC) curve analysis for severe postoperative complications (Figure 4), patients were divided into two groups: the NPS score 0–1 and 2–4 groups. Postoperative complications were compared between the two groups. Although the incidence of overall complications did not show a significant difference between these groups (57.6% vs. 52.1% and *p* = 0.35), the incidence of CD grade ≥ IIIa postoperative complications was significantly higher in the NPS score 2–4 group than in the NPS score 0–1 group (20.9% vs. 11.5% and *p* = 0.03) (Table 3). Moreover, comparing the incidence of each postoperative complication, the incidences of PHLF (20.5% vs. 11.5% and *p* = 0.04) and ascites (7.6% vs. 1.0% and *p* = 0.01) were significantly higher in the NPS score 2–4 group (Table 3). In contrast, there were no significant differences in the incidences of posthepatectomy bile leakage (*p* = 0.48), pleural effusion (*p* = 0.92), abdominal infection (*p* = 0.95), and incisional surgical site infection (*p* = 0.51) (Table 3).

**TABLE 3:** Summary of postoperative complications.

	NPS score 0–1		NPS score 2–4		*p* value
	*n* = 96		*n* = 278		
Mortality	1	(0.4)	0	(0)	1.00
Morbidity					
All grade	50	(52.1)	160	(57.6)	0.352
Clavien–Dindo classification grade ≥ IIIa	11	(11.5)	58	(20.9)	0.033^a^
Posthepatectomy liver failure	11	(11.5)	57	(20.5)	0.039^a^
Posthepatectomy bile leakage	8	(8.3)	30	(10.8)	0.483
Ascites	1	(1.0)	21	(7.6)	0.007^a^
Pleural effusion	10	(10.4)	30	(10.8)	0.918
Incisional surgical site infection	12	(12.5)	28	(10.1)	0.513
Intraperitoneal infection	5	(5.2)	14	(5.0)	0.947

*Note*: Figures in parentheses are percentage unless otherwise specified.

Abbreviation: NPS, Naples prognostic score.

^a^ *p* < 0.05.

Multivariate analysis identified an NPS score of 2–4 as an independent risk factor for the incidence of CD grade ≥ IIIa postoperative complications (OR, 2.05; 95% CI, 1.01–4.18; and *p* = 0.05) along with an indocyanine green retention rate at 15 min >11.8% (OR, 2.35; 95% CI, 1.31–4.25; and *p* < 0.01) and diabetes mellitus status (OR, 2.04; 95% CI, 1.18–3.54; and *p* = 0.01) (Table 4).

**TABLE 4:** Univariate and multivariate analyses of risk factors for severe complications.

Variables	Univariate			Multivariate		
	Odds ratio	95% CI	*p* Value	Odds ratio	95% CI	*p* Value
Age						
<60	Ref			Ref		
≥60	2.45	0.94–6.40	0.067	2.24	0.84–5.99	0.109
Diabetes mellitus						
No	Ref			Ref		
Yes	2.09	1.23–3.56	0.007^a^	2.04	1.18–3.54	0.011^a^
ICG retention rate at 15 min						
<11.8%	Ref			Ref		
≥11.8%	2.39	1.39–4.10	0.001^a^	2.35	1.31–4.25	0.004^a^
Anatomic resection						
No	Ref			Ref		
Yes	1.85	1.04–3.29	0.036^a^	1.29	0.74–2.61	0.301
NPS score						
0–1	Ref			Ref		
2–4	2.04	1.02–4.07	0.044^a^	2.05	1.01–4.18	0.048^a^

Abbreviations: CI, confidence interval; ICG, indocyanine green; NPS, Naples prognostic score.

^a^ *p* < 0.05.

## 4 DISCUSSION

The results of the present study emphasize that the NPS can predict the prognosis and postoperative complications of HCC after hepatectomy with high accuracy and NPS, along with portal vein invasion and multiple tumors, was found to be an independent prognostic factor. Moreover, the prognostic value of the NPS is more useful in patients with UICC stage I or II HCC.

The NPS is an IBPS developed as a prognostic marker for colorectal cancer by Galizia et al.^10^ The NPS includes factors that reflect nutritional status (albumin and TC) and inflammation (NLR and LMR) and has been reported to be a novel prognostic marker of various types of cancer such as colorectal,^10^, ^15^ lung,^8^, ^14^ and esophageal^12^, ^13^ cancers. To our knowledge, only one study has focused on its usefulness in HCC,^9^ in which Xie et al. reported the predictability of the NPS for the OS and RFS and the superiority of NPS over NLR, LMR, systemic inflammation score, prognostic nutritional index, controlling nutritional status score in 5‐year OS, and time‐dependent ROC for OS. The result of our study supports the study of Xie et al. regarding the usefulness of the NPS for prognostic implication. However, their study did not examine the relationship between postoperative complications and the NPS or explore in which patients' NPS could predict prognosis with great accuracy. In the present study, we revealed that NPS was one of the independent prognostic factors and the prognostic predictability of the NPS is superior in patients with UICC stage I–II cancers by subgroup analysis. Various factors, including size, number, tumor markers, and vascular invasion, are well‐known prognostic indicators for HCC, and in this study, multiple tumors and portal vein invasion were extracted as independent prognostic factors along with NPS. Notably, the HR for portal vein invasion was higher than that for NPS. However, the preoperative diagnosis of microscopic portal vein invasion is challenging, making NPS advantageous for identifying high‐risk groups preoperatively. Moreover, identifying patients with a poor prognosis without highly advanced cancers is generally considered difficult and the NPS is valuable in this regard. In addition, patients with a high NPS presented with a significantly high incidence rate of severe complications. To our knowledge, this is the first study to explore this association in hepatectomy.

The underlying mechanisms of prognostic prediction by the NPS remain controversial; however, the mechanism has been discussed in several reports.^7^, ^8^, ^9^ The NPS may reflect the tumor microenvironment (TME) immune system.^8^ The main reason for these claims is that the NPS is an IBPS that integrates the serum albumin and TC levels and lymphocyte, neutrophil, and monocyte counts, all of which are associated with the antitumor immune system. Albumin and TC levels are well‐known indicators of malnutrition. Furthermore, they also have functions associated with tumor immunity such as the reflection of chronic inflammation by the albumin level^21^, ^22^ and the maintenance of the immune cell cytoskeleton by the TC.^23^, ^24^ Numerous studies have highlighted the functions of lymphocytes, neutrophils, and monocytes in TME. Lymphocytes mainly function as tumor suppressors, whereas neutrophils can promote tumor progression directly^25^ or suppress lymphocyte function via inflammation^26^ and monocytes differentiate into tumor‐associated macrophages.^27^ Among these immune cells, tumor‐infiltrating lymphocytes (TILs) play a critical role in tumor immunity.^28^, ^29^ TILs elicit an immune response against tumor cells via the perforin, granzyme B, FAS‐FAS ligand axis, and cytokines and induce apoptosis of tumor cells. Furthermore, TILs activate antitumor responses in the TME by secreting cytokines. Tanaka et al. demonstrated that peripheral blood lymphocyte counts correlated with TIL counts,^30^ and the inclusion of the two items assessing the lymphocyte count (NLR and LMR) in the NPS might be responsible for its high prognostic predictability. Moreover, antitumor immune mechanisms are closely related to tumor progression and immune evasion advances in tandem with tumor progression.^25^ These findings are consistent with those of the present study and support the usefulness of the NPS in predicting the prognosis of patients with UICC stage I–II HCC. Furthermore, the NPS is valuable in predicting complications because it serves as an indicator of inflammation, malnutrition, and immunosuppression as mentioned.

Based on these findings, certain strategies for utilizing the NPS in clinical practice can be anticipated. First, NPS might be utilized as a marker for neoadjuvant chemotherapy (NAC) and adjuvant chemotherapy (AC), including regimens that incorporate immunotherapy, which hold promise for clinical application in the near future. Although additional data collection is required, administering NAC or AC in patients with high NPS may enhance their prognosis. Second, modifying the surgical method or intensifying postoperative management in patients with an NPS score of 2–4 could improve surgical outcomes, as these patients experienced relatively frequent severe complications.

The present study has some limitations. First, this was a single‐center retrospective study with a limited number of patients and various potential bias and confounding factors cannot be excluded. Second, the results of this study were based on data from patients with HCC from 2007 to the present and might not represent real‐world data from the era of immune checkpoint blockade therapy. Therefore, future large‐scale prospective multicenter studies with meticulous regulation of all the identified covariates are needed. Furthermore, a long‐term follow‐up period is essential for evaluating the predictive ability of the NPS over time. Third, this study included no data regarding differences in pathological or molecular biological characteristics according to the NPS. Research on these characteristics is necessary to explore the underlying mechanisms driving the prognostic implications of NPS, and prospective validation analyses should also be conducted to clarify these relationships.

In conclusion, the NPS is an effective predictor of short‐ and long‐term outcomes after hepatectomy in patients with HCC and may be a potentially superior prognostic marker even in patients with nonadvanced HCC.

## AUTHOR CONTRIBUTIONS

Kiyotaka Hosoda, Akira Shimizu, Koji Kubota, Tsuyoshi Notake, Noriyuki Kitagawa, and Yuji Soejima were involved in the study design and data interpretation. Kiyotaka Hosoda drafted the manuscript. Kiyotaka Hosoda, Takahiro Yoshizawa, Hiroki Sakai, Hikaru Hayashi, and Koya Yasukawa collected and analyzed the data. Yuji Soejima supervised the study. All authors critically revised the manuscript, commented on drafts of the manuscript, and approved the final report.

## CONFLICT OF INTEREST STATEMENT

The authors declare that they have no conflicts of interest.

## ETHICS STATEMENT

The protocol for this research project has been approved by a suitably constituted Ethics Committee of the institution and it conforms to the provisions of the Declaration of Helsinki (the Shinshu University Hospital Ethics Committee, Approval No. 5244).

## INFORMED CONSENT

An opt‐out procedure was applied to the website to obtain informed consent and patients who refused to participate were excluded.

## SOURCES OF SUPPORT

None.

## Supporting information

Supporting Information S1

Table S1

Figure S1

## Data Availability

The data that support the findings of this study are available from the corresponding author upon reasonable request.
