# Supplementary material for: Clinical significance of the Naples prognostic score in predicting short‐ and long‐term postoperative outcomes of patients with hepatocellular carcinoma
Source: World J Surg. 2024 Dec 4;49(2):502–11. doi: 10.1002/wjs.12448 (PMC11798678; doi:10.1002/wjs.12448)
Supplement: Supplementary file 1 — Supporting Information S1 [file WJS-49-502-s001.docx]

Fig. S1 Kaplan–Meier curves for the OS and RFS of the participants stratified with a single tumor smaller than 5 cm by high- and low-NPS. The OS (a) and RFS (b) of the high-NPS group are significantly lower than that of the low-NPS group (OS, P = 0.01; RFS, P = 0.02). OS, overall survival; RFS, recurrence-free survival; NPS, Naples prognostic score
