# Supplementary material for: Clinical significance of the Naples prognostic score in predicting short‐ and long‐term postoperative outcomes of patients with hepatocellular carcinoma
Source: World J Surg. 2024 Dec 4;49(2):502–11. doi: 10.1002/wjs.12448 (PMC11798678; doi:10.1002/wjs.12448)
Supplement: Supplementary file 2 — Table S1 [file WJS-49-502-s003.docx]

TABLE S1. Uni- and multivariate analyses of prognostic factors for recurrence-free survival

| Variables | Univariate | | |  | Multivariate | | |
| --- | --- | --- | --- | --- | --- | --- | --- |
|  | Hazard ratio | 95% CI | *P* value |  | Hazard  Ratio | 95% CI | *P* value |
| Tumor diameter | | | | | | | |
| < 5 cm | Ref |  |  |  | Ref |  |  |
| ≥ 5 cm | 1.87 | 1.39-2.49 | <.001* |  | 1.64 | 1.22-2.20 | .002 |
| Multiple tumor | | | | | | | |
| No | Ref |  |  |  | Ref |  |  |
| Yes | 2.04 | 1.52-2.72 | <.001* |  | 1.88 | 1.39-2.52 | <.001* |
| Alpha-fetoprotein | | | | | | | |
| < 10 ng/ml | Ref |  |  |  | Ref |  |  |
| ≥ 10 ng/ml | 1.94 | 1.48-2.53 | <.001* |  | 1.65 | 1.25-2.18 | <.001* |
| Portal vein invasion | | | | | | | |
| No | Ref |  |  |  | Ref |  |  |
| Yes | 2.08 | 1.55-2.76 | <.001* |  | 1.68 | 1.24-2.26 | <.001* |
| NPS | | | | | | | |
| Low-NPS | Ref |  |  |  | Ref |  |  |
| High-NPS | 1.34 | 1.01-1.77 | .046* |  | 1.34 | 1.00-1.77 | .049* |

Abbreviations: CI, confidence interval; NPS, Naples prognostic score

**P* < .05
