# Supplementary figures and images for: Clinical significance of the Naples prognostic score in predicting short‐ and long‐term postoperative outcomes of patients with hepatocellular carcinoma
Source: World J Surg. 2024 Dec 4;49(2):502–11. doi: 10.1002/wjs.12448 (PMC11798678; doi:10.1002/wjs.12448)

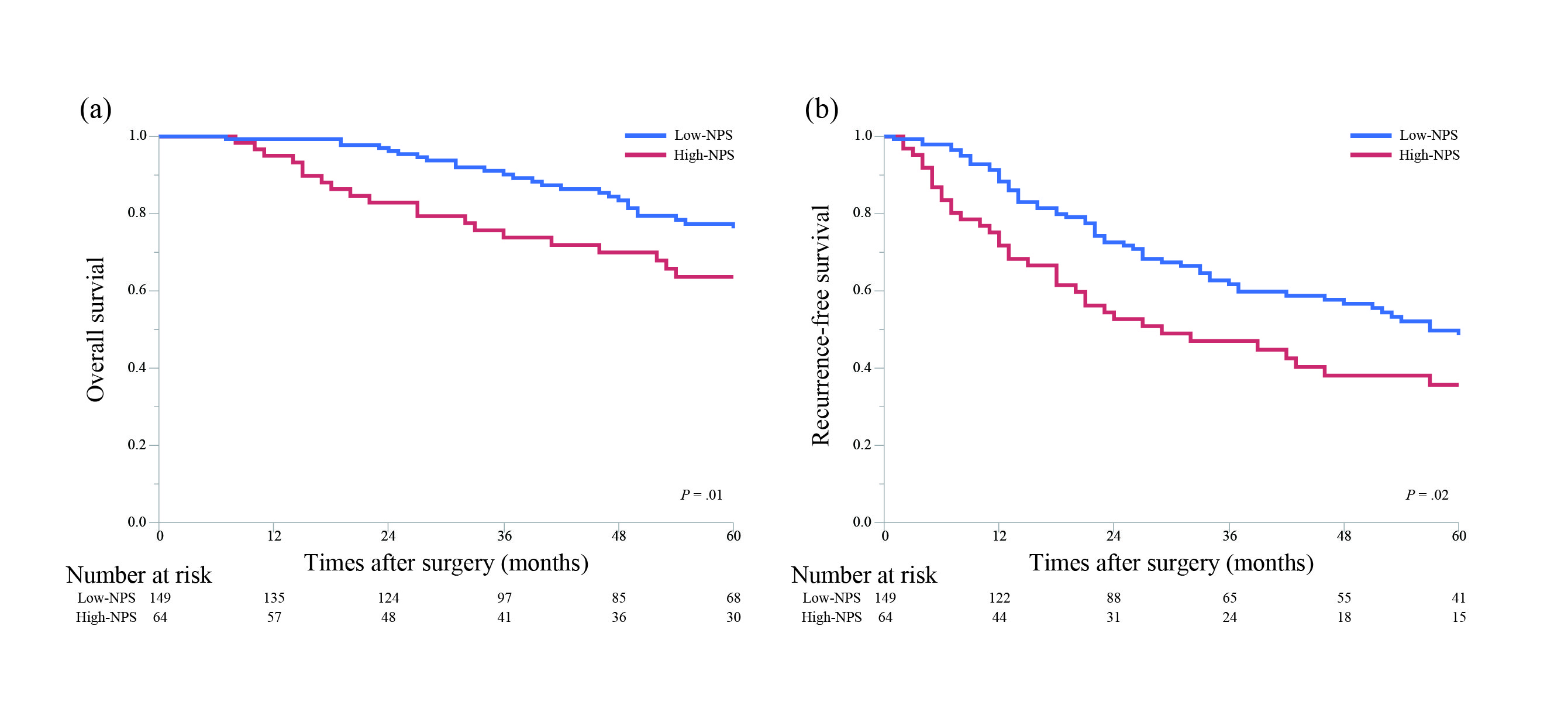

Supplement: Supplementary file 3 — Figure S1 [file WJS-49-502-s002.jpg]
